# Occurrence and Risk Assessment of Pesticides, Phthalates, and Heavy Metal Residues in Vegetables from Hydroponic and Conventional Cultivation

**DOI:** 10.3390/foods13081151

**Published:** 2024-04-10

**Authors:** Shanshan Chen, Chunxia Yao, Jiaxin Zhou, Haiyao Ma, Jing Jin, Weiguo Song, Zhenpeng Kai

**Affiliations:** 1 Institute of Agro-Food Standards and Testing Technology, Shanghai Academy of Agricultural Sciences, Shanghai 201403, China; shanshanchen2013@saas.sh.cn (S.C.); chunxiayao2007@saas.sh.cn (C.Y.); zhou_jx@126.com (J.Z.); 2 School of Chemical and Environmental Engineering, Shanghai Institute of Technology, Shanghai 201418, China; quantumcat@126.com (H.M.); jinjing283767@163.com (J.J.)

**Keywords:** risk assessment, hydroponic, vegetable, pesticide residues, phthalates, heavy metals

## Abstract

Hydroponic cultivation of fresh produce is gaining popularity worldwide, but few studies have provided a comparative assessment of hydroponic and conventional soil-based vegetables. In this study, we analyzed a series of hazardous chemicals, including 120 pesticides, 18 phthalates (PAEs), and 2 heavy metals (lead and cadmium) in four vegetable commodities (lettuces, celeries, tomatoes, and cucumbers) from hydroponic and conventional soil-based cultivation. Our study showed that at least one pesticide was present in 84% of the conventionally grown samples, whereas only 30% of the hydroponic samples contained detectable pesticide residues. Regarding the total PAE concentrations, there was no significant difference between conventional and hydroponic vegetables. The lead and cadmium residues in conventionally cultivated vegetables were significantly higher than in those produced from hydroponic cultivation. Lead is the primary heavy metal pollutant across all vegetable samples. The hazard index (HI) values of the hydroponic and conventional vegetables were 0.22 and 0.64, respectively. Since both values are below one, the exposure to these hazardous chemicals through consumption of the studied vegetables may not pose a significant health risk. The HI values also suggested that the health risks of eating hydroponic vegetables are lower than for conventional soil-based vegetables.

## 1. Introduction

Hydroponics is the cultivation of plants without using soil, which instead uses water to grow plants in mineral nutrient solutions [1]. It is an effective food production technique that could feed an expanding population with declining land resources. Commercial hydroponic farms have been developed in many countries around the world. The method is expected to grow at a global rate of 18.8% from 2017 to 2023, reaching USD 490.5 million in the global hydroponic market by 2023 [2]. There are many advantages of growing vegetables under hydroponic cultivation over conventional soil-based cultivation [3]. Hydroponics does not require a large land area and does not rely on seasons, in contrast to soil-based culture. The achievement of high yields, good quality, and healthy vegetables are possible with hydroponics due to the precise control of water, nutrition, and all growing conditions [4]. Hydroponic vegetables are generally protected from diseases that spread through the soil or pests in the open field. Therefore, they require fewer chemicals to control pests and are safer than conventionally grown vegetables in terms of possible chemical contamination [3]. Various commercial vegetables can be grown using hydroponics, such as leafy greens, tomatoes, cucumbers, and peppers. As the cultivation process is more controlled, hydroponic vegetables are often less affected by pests and diseases. In addition, vegetables that are cultivated in soils may uptake organic pollutants and heavy metals from the contaminated soil, irrigation water, and biomass fertilizers through their roots. Some studies have examined the nutritional quality of agricultural products and the pesticide residues from organic vs. conventional farming [5,6]. However, the comparative research on the risks of vegetables growing in hydroponic and soil cultures is limited.

Pesticide exposure has been shown to be associated with a wide range of health problems. It is very important to elucidate pesticide residues in plants for assessing the impact of pesticide contamination on food safety [7]. The greenhouse where crops are grown in controlled environments with plastic shed and film, including hydroponic cultivation, has been essential for commercial vegetable production in China. However, vegetables can be contaminated by using plastic facilities during their growth. The chemicals phthalates (the esters of phthalic acid, PAEs) are mainly used as plasticizers to increase a plastic materials’ flexibility, transparency, durability, and longevity. Because of the estrogenic effects of the phthalate contaminants and the potentially harmful effects on the development of growing infants, PAEs are considered an environmental endocrine-disrupting chemical. PAEs are considered to be reproductive toxicants, teratogens, and carcinogens [8]. Endocrine-disrupting chemicals (EDCs) are a growing public health concern worldwide. PAEs, lead, cadmium, organochlorine pesticides, etc., are all EDCs [9]. Recently, residues of EDCs in vegetables are receiving intensive attention from the public. 

Our work was initiated to investigate and document the occurrence and levels of various pesticides, PAEs, and heavy metals in vegetables from hydroponic and conventional soil-based cultivation. Furthermore, we performed risk assessments of pesticides, PAEs, and heavy metals in 177 lettuce, celery, tomato, and cucumber samples from hydroponic and conventional cultivation by calculating the estimated daily intake (EDI) target hazard quotient (THQ) and the hazard index (HI).

## 2. Materials and Methods

### 2.1. Chemicals

In total, 120 pesticides, 18 PAEs, and 2 heavy metals were analyzed in this study. All the pesticide, PAE (shown in SI Tables S1 and S2), and heavy metal (lead and cadmium) standards were purchased from Dr. Ehrenstorfer GmbH (Augsburg, Germany) and AccuStandard, Inc. (New Haven, CT, USA), with a purity of ≥95% (typically >99%). Acetonitrile, methanol, acetone, and hexane were of HPLC grade and were obtained from Fisher Scientific (Fair Lawn, NJ, USA). Individual stock standard solutions (1000 mg/L) were prepared in acetone, methanol, or hexane, depending on the solubility of each standard. All the solutions were stored at −20 °C in order to improve their stability. Extraction salt packets containing 4 g of magnesium sulfate, 1 g of anhydrous sodium, 1 g of sodium citrate, 0.5 g of sodium hydrogen citrate, and purification sorbents consisted of primary and secondary amine (PSA), graphitized carbon black (GCB), and magnesium sulfate. All were purchased from Agilent Technologies (Lake Forest, CA, USA).

### 2.2. Sample Collection and Preparation

During the 2022–2023 harvest season, 177 samples of fresh vegetables (lettuces, celeries, tomatoes, and cucumbers) were purchased directly from hydroponic vegetable factories and conventional farms in the suburban districts of Shanghai. The sample number of each vegetable grown under different cultivation conditions is shown in Table 1. The varieties of fresh lettuce samples (*n* = 58, 32 hydroponic and 26 conventional) included romaine (*n* = 29), iceberg (*n* = 15), and Boston (*n* = 14). Both Chinese celery (*n* = 19) and American celery (*n* = 16) samples were collected from hydroponic (*n* = 15) and conventional (*n* = 20) farming. The varieties of tomato samples (*n* = 42, 20 hydroponic and 22 conventional) included Roma (*n* = 12), cherry (*n* = 19), and round (*n* = 11) tomatoes. Two types of cucumbers (Holland cucumber and Chinese cucumber) were collected from hydroponic (*n* = 19) and conventional (*n* = 23) farming. Among all 177 vegetable samples, 86 samples were from hydroponic cultivation, and the other 91 were from conventional soil-based cultivation.

Plastic containers and apparatus were not used to avoid possible PAE pollution during this study’s sample collection, preparation, and analytical procedure. The vegetables were randomly collected using pre-cleaned stainless steel scissors, placed into pre-cleaned aluminum foil bags, and then immediately transported to the laboratory. In the lab, each sample was ground with a blender and stored at −20 °C until analysis.

### 2.3. Determination of Contaminants

Contaminants including pesticides, phthalates, and heavy metals were determined in multiclass analytical laboratories complying with the ISO-IEC 17025 requirements [10]. Approximately 10.0 g of vegetable sample was weighed into a 50 mL centrifuge tube for pesticide residue analysis. An automatic single-step quick, easy, cheap, effective, rugged, and safe (QuEChERS) sample preparation device was used for the analysis of pesticide residues in vegetables, combined with gas chromatography–tandem mass spectrometry (GC-MS/MS) [11] and ultra-performance liquid chromatography–tandem mass spectrometry (UPLC-MS/MS), as reported by He et al. [12]. The GC-MS/MS system (Thermo Fisher Scientific, Waltham, MA, USA) consisted of a Thermo Trace series GC coupled with an AI/AS 1310 autosampler and a TSQ 9000 triple quadrupole mass spectrometer. The UPLC-MS/MS system consisted of Waters ACQUITY UPLC I-Class (Waters Corp., Milford, MA, USA), coupled with AB SCIEX TRIPLE QUADTM 5500 mass spectrometer from AB Sciex (Framingham, MA, USA). Analyte-specific MS/MS conditions and LC retention times for LC-amenable analytes are shown in Table S3. The optimal two ion transitions (primary and secondary transitions of a precursor to product) for the multiple reaction monitoring (MRM) of each pesticide were determined via collision tests (SI Table S4). The local government of Shanghai publishes a list of recommended pesticides every year. All farms, including hydroponics and conventional culture, are subject to these regulations. Therefore, both hydroponic and conventional vegetable samples were assessed for these 120 pesticide residues. PAE testing was performed by solid-phase extraction and GC-MS/MS analysis, as reported by Wang et al. [13]. First, 10.0 g of vegetable sample was weighed into a glass centrifuge bottle and mixed with 20 mL acetone/hexane (1:1 *v*/*v*). The mixture was left overnight and then ultrasonicated for 30 min. The extraction procedure was described in detail by Wang et al. [13]. The parameters and collision energy of parent ions and the quantification and identification of ions for PAEs are listed in Table S5. Heavy metals were determined using inductively coupled plasma mass spectrometry (ICP-MS) (Agilent Technologies, 7500 CX, USA). The vegetables were prepared by vacuum freeze-drying technology. The sample preparation and analysis procedures for heavy metal determination were described in detail by Zhang et al. [14].

### 2.4. Data Analysis and Statistics

The residue data were compiled in Microsoft Office Excel 2010. Statistical analyses were performed with GraphPad Prism version 5.0. A value of 0.05 was used as the threshold for significance. Differences between hydroponic and conventional samples regarding contaminant frequencies of detection were evaluated using the χ2 (chi-square) test.

### 2.5. Risk Assessment Calculation

The estimated dietary intake (EDI) depends on the concentration of individual contaminants in the four vegetables and the daily consumption of vegetables. The EDI (μg. kg BW^−1^. d^−1^) was calculated following Equation (1):

EDI = C × Con/Bw(1)

where C (μg kg^−1^) is the average concentration of a given contaminant in the collected vegetable samples, Con (kg. DAY^−1^) is the average daily consumption of vegetables in the region, and Bw (kg person^−1^) represents body weight. The values of contaminants below the LOQ were not included in the calculation of mean values. A daily dose of 242.0 g [15] was used for the risk assessment calculation from the consumption of vegetables in China. An average adult body weight of 60 kg was applied in this calculation [16].

Non-carcinogenic health risks through consuming contaminated vegetables for residents were estimated according to the target hazard quotient (THQ) [17]. The THQ through vegetable consumption can therefore be assessed based on the food chain and acceptable daily intake (ADI), tolerable daily intake (TDI), or an oral reference dose (RfD) for each contaminant. The equations used were as follows:

THQpesticides = EDI/ADI(2)

THQPAEs = EDI/TDI(3)

THQheavy metals = EDI/RfD(4)

THQpesticides represents the THQ values of pesticides, THQPAEs is the THQ values of PAEs, and THQheavy metals is the THQ values of heavy metals. The applied ADI was obtained from GB 2763-2021 [18] of China, and the applied TDI and RfD were obtained from the US EPA. Equation (2) was used to calculate the risk assessment of pesticides. Equations (3) and (4) were applied for the risk assessment of PAEs and heavy metals from vegetables. 

The hazard index (HI) measures the potential risk of adverse health effects from a mixture of chemical constituents [16]. It is a widely used method for mixture risk assessment (MRA). The HI through average daily consumption of food was calculated as follows:

(5) $$\mathrm{HI}=\sum_{i=1}^{n}\mathrm{THQi}$$

## 3. Results and Discussion

### 3.1. Pesticide Residue Comparison between Hydroponic and Conventional Vegetables

In this study, 53% of all the 177 samples contained at least one pesticide. While 84% of the conventionally grown samples carried at least one pesticide, only 30% of the hydroponic samples had pesticide residues. We found a significant difference in the detection rate of pesticide residues between conventional and hydroponic vegetables (*p* < 0.0001). The residue of 29 pesticides (15 insecticides, 12 fungicides, 1 herbicide, and 1 plant growth regulator) was found in all vegetable samples. Neonicotinoids were the most recurrently detected class, with 29% of the samples presenting levels above the LOQ. The most frequently detected pesticides were acetamiprid (19%), followed by chlorantraniliprole (10%), dimethomorph (9%), difenoconazole (8%), carbendazim (7%), chlorfenapyr (7%), and imidacloprid (6%). Only acetamiprid and chlorantraniliprole were detected across all four commodities. Our results agree with the study finding that mixed insecticides and fungicides are widely used for vegetable protection [6,19].

**Figure 1:**
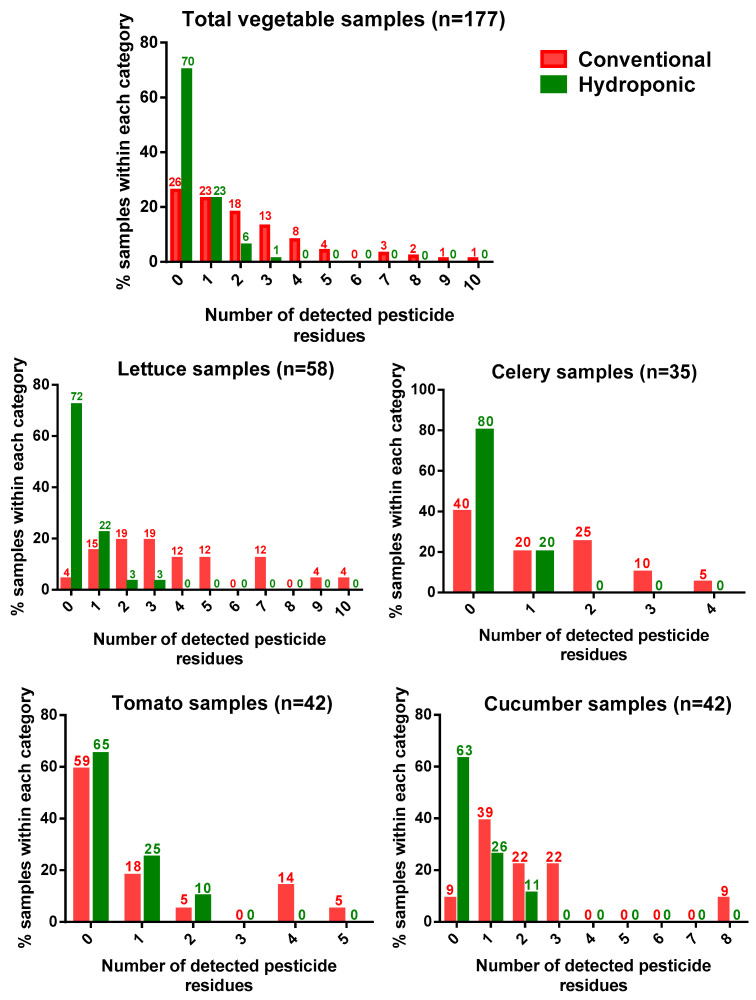
Percentage of samples (%) according to the number of detected pesticide residues for conventional and hydroponic vegetables.

Remarkable differences were also observed in the detection frequencies of pesticide residues between conventional and hydroponic samples. Based on the overall dataset (n = 177), 51% of the conventionally grown samples contained two or more pesticide residues, compared to 7% in the hydroponic samples (Figure 1). The frequency of detection of insecticides was significantly higher for conventional samples (57%) than that of hydroponic samples (12%). Similarly, the proportion of fungicide levels > LOQ from conventional samples (40%) was significantly higher than that from hydroponic ones (5%). One herbicide, pendimethalin, and a plant growth regulator, paclobutrazol, were detected only in conventionally cultivated samples. The summed pesticide levels (ΣPesticides) ranged between <LOQ and 2.22 mg kg^−1^ for conventional samples and <LOQ and 0.44 mg kg^−1^ for hydroponic samples. It has been reported that among the fresh produce grown in Canada, the proportion of insecticides at detectable levels (>LOQ) in samples from conventionally cultivated produce was significantly higher than in those from organic practice [6]. Our results show that, like organic produce, hydroponic vegetables have fewer pesticide residues than conventional soil-based vegetables. 

In lettuce, 59% of the samples tested positive for at least one pesticide. In total, 13 insecticides and 10 fungicides were detected across all the lettuce samples. The most frequently detected pesticide in the lettuces was acetamiprid (24%), followed by chlorantraniliprole (19%), difenoconazole (19%), and chlorfenapyr (19%). A significant difference in the detection rate of pesticide residues between conventional and hydroponic lettuce was found (*p* < 0.0001) (Figure 1). We found that 96% of lettuce samples produced from conventional cultivation carried at least one pesticide, whereas only 28% of the hydroponic lettuce samples contained pesticide residues. In the conventionally grown lettuce samples, the average number of pesticide detection was four, and the maximum number of pesticide residues in a single sample was ten. When looking into the conventional lettuce samples, five samples (19%) had pesticide levels over China’s maximum residue limit (MRL). In these samples, three insecticides (acetamiprid, chlorfenapyr, and cyromazine) and one fungicide (boscalid) exceeded their maximum residue limits. The pesticide residues in the hydroponic lettuce samples were quite different. Only two hydroponic samples (6%) were found with multiple pesticide residues, and the maximum number of pesticide residues in a single sample was three. None of the hydroponic lettuce samples contained pesticide residues over the MRL. 

The celery samples contained fewer pesticide residues than the lettuce samples did. In total, five insecticides (imidacloprid, acetamiprid, chlorpyrifos, cyhalothrin, and chlorfenapyr) and four fungicides (carbendazim, azoxystrobin, dimethomorph, and difenoconazole) were detected in all the celery samples. All nine pesticides were detected in the conventional celery samples, whereas the celery samples produced from hydroponic cultivation were identified to only contain two pesticides (chlorpyrifos and dimethomorph). A significant difference in the detection rate of pesticide residues between conventional and hydroponic celery was found (*p* < 0.0001) (Figure 1). In total, 60% of conventional celery samples carried at least one pesticide, and 40% had multiple pesticide residues. This result follows previous research on celery in China [20,21]. In our study, the hydroponic celery samples had a pesticide detection rate of 20%, and only three contained one pesticide residue. 

In total, six insecticides and two fungicides were detected in all the tomato samples. The most frequently detected pesticide in the tomato samples was acetamiprid (19%), followed by chlorantraniliprole (17%) and carbendazim (12%). We found that 41% of the tomato samples produced from conventional cultivation had at least one pesticide, whereas 35% of the hydroponic tomato samples contained pesticide residues. The detection rates of multiple pesticide residues in conventional and hydroponic tomato samples were 24% and 10%, respectively. There was a significantly different detection rate of pesticide residues between conventional and hydroponic tomato samples (*p* < 0.0002, Figure 1). He et al. showed that the environmental impact index for organic tomato production was 54.87% lower than for conventional tomato production in urban greenhouses of Beijing city [22]. Like organic tomatoes, hydroponic cultivation can also reduce pesticide residues and other toxicity impacts in tomatoes.

Three insecticides, five fungicides, one herbicide, and one plant growth regulator were detected in the cucumber samples. In total, 67% of the samples tested positive with at least one pesticide. The most frequently detected pesticide in the cucumber samples was acetamiprid (21%), followed by dimethomorph (14%) and boscalid (14%). Golge et al. demonstrated that the residues found in cucumbers in Turkey belonged to the groups of fungicides and insecticides [23]. The most frequently detected residues in their cucumber samples were propamocarb, acetamiprid, and dimethomorph, which is similar to our results. A significant difference in the detection rate of pesticide residues between conventional and hydroponic cucumbers was found (*p* < 0.0001, Figure 1). We found that 91% of the cucumber samples produced from conventional cultivation contained at least one pesticide, whereas only 37% of the hydroponic cucumber samples exhibited pesticide residues. The detection rates of multi-pesticide residues in conventional and hydroponic cucumber samples were 52% and 11%, respectively.

Pesticide residue is one of the most critical issues in food safety. Hydroponics is a technique of growing plants in a nutrient solution and in the complete absence of soil, which leaves no chance of soil-borne diseases, insects, or pest infections affecting the plants, thereby reducing or eliminating the use of pesticides and their resulting health effects [3]. Additionally, hydroponics requires less growing time than field cultivation or protected environments such as a greenhouse. The shortened growing time enables hydroponic vegetables to reduce pesticide use to control pests, weeds, and diseases [3,24]. Similarly, our studies have demonstrated and validated that the pesticide residues in two leafy vegetables (lettuce and celery) and two fruit vegetables (tomato and cucumber) from hydroponic cultivation were significantly lower than in those from conventional cultivation. Although hydroponic cultivation is an advantageous technique, the application of pesticides is inevitable due to the invasion of pathogens and pests outside the hydroponic system and water-borne diseases that can spread quickly from one plant to another [25]. Hydroponic vegetables are considered organic food. Studies agree in finding organic food much less contaminated by pesticides and with residues of much lower toxicity compared to those found in conventional foods [26]. Complete environmental control, disinfection of seeds, and irrigation water may further reduce pesticide application.

### 3.2. Comparison of the Phthalate Residues between Hydroponic and Conventional Vegetables

**Figure 2:**
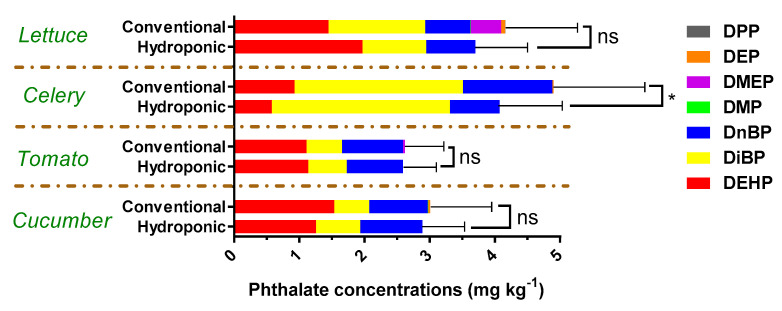
Concentrations of DEPH, DEP, DiBP, DMEP, DMP, DnBP, DPP, and ΣPAEs in conventional and hydroponic vegetables. Values represent means ± SDs, * *p* < 0.05. Statistical significance was assessed with *t*-tests.

The individual concentrations of 18 PAEs in the four vegetables produced from conventional and hydroponic cultivation are shown in Table 2 and Table 3, respectively. PAEs were detected in all samples. Figure 2 shows that the total PAE concentrations in hydroponic vegetables were slightly lower than those in the conventional samples. However, there was no significant difference in the total amount of PAEs between the conventional and hydroponic cultivation samples (except for the celery sample). A significant difference in the total PAE concentrations between the hydroponic and conventional celery was found (*p* = 0.042). Di-iso-butyl phthalate (DBP), Bis-2-Ethylhexyl phthalate (DEHP), and di-n-butyl phthalate (DnBP) were detected in all samples, with higher frequencies of detection. The detectable rates of DiBP, DEHP, and DnBP were 82.5, 81.9, and 81.4%, respectively. The mean concentration of DiBP, DEHP, and DnBP in all the samples was 1.37, 1.45, and 0.92 mg kg^−1^. Our results were consistent with those of many other research groups that have studied the contamination of PAEs in China [13,27,28].

Seven PAEs were detected in the conventionally grown lettuce samples (Table 3). The sum of the seven PAE (∑7PAEs) concentration in the conventional lettuce samples ranged from 2.47 to 5.85 mg kg^−1^, with a median of 2.92 mg kg^−1^. Concerning individual PAEs, DiBP, DEHP, DnBP, and DMEP were found in each conventional lettuce sample, while the detection frequencies of DEP, DMP, DHP, and DPP were 92.3, 84.6, and 65.4%, respectively. DiBP exhibited the highest concentration among the analyzed PAE congeners, followed by DEHP, DnBP, and DMEP, with average residue levels of 1.48, 1.44, 0.69, and 0.47 mg kg^−1^, respectively (Figure 2). Only three PAEs (DiBP, DEHP, and DnBP) were detected in the hydroponic lettuce samples. The sum of the three PAE (∑3PAEs) concentrations in the hydroponic lettuces ranged from 2.51 to 4.89 mg kg^−1^, with a median of 2.78 mg kg^−1^.

Among the 18 PAEs analyzed in this study, DiBP, DEHP, and DnBP were present in all celery samples from both conventional and hydroponic cultivation (Figure 2). DEP was also detected in the conventional celery samples with an 80% detection frequency. The average total PAE value in the conventional and hydroponic celery samples was 5.05 and 4.07 mg kg^−1^, respectively. Among all the analyzed PAE congeners in all celery samples from both conventional and hydroponic cultivation, DiBP exhibited the highest concentration, accounting for more than half of the total appearance of PAEs (Figure 2). Other major congeners were DnBP and DEHP. In the conventional celery samples, the average concentrations of DiBP, DnBP, DEHP, and DEP were 2.58, 1.37, 0.93, and 0.17 mg kg^−1^, respectively. Similarly, the average DiBP, DnBP, and DEHP residue levels in the hydroponic samples were 2.73, 0.76, and 0.57 mg kg^−1^, respectively.

Interestingly, the PAEs residue levels in the fruit vegetables were lower than in the leafy vegetables (Figure 2). The average total PAE value in the conventional and hydroponic tomato samples was 2.62 and 2.54 mg kg^−1^, respectively. DEHP, DnBP, DiBP, and DEP were detected in the conventional tomato samples with a frequency of 54.5, 50, 45.5, and 45.5%, respectively. DEHP exhibited the highest concentration in the conventional tomato samples, followed by DnBP, DiBP, and DEP, whose average residue levels were 1.11, 0.94, 0.55, and 0.03 mg kg^−1^, respectively. In the hydroponic tomato samples, only DEHP, DnBP, and DiBP were observed. The frequencies of detection followed the order of DEHP (80%), DnBP (75%), and DiBP (75%), with the average concentrations being 1.13, 0.81, and 0.59 mg kg^−1^, respectively.

Five PAEs (DEHP, DnBP, DiBP, DEP, and DMP) were detected in the conventionally grown cucumber samples, whereas only three PAEs (DEHP, DnBP, and DiBP) were observed in the hydroponic cucumbers. The average total PAE values in the conventional and hydroponic cucumber samples were 3.01 and 2.89 mg kg^−1^, respectively (Figure 2). In the cucumber samples, both from conventional and hydroponic cultivations, DEHP also exhibited the highest concentration and frequencies of detection, followed by DnBP and DiBP. In the conventional cucumber samples, the average concentrations of DEHP, DnBP, DiBP, DEP, and DMP were 1.54, 0.90, 0.54, 0.03, and 0.01 mg kg^−1^, respectively. Similarly, the average residue levels of DEHP, DnBP, and DiBP in the hydroponic samples were 1.26, 0.95, and 0.68 mg kg^−1^, respectively.

Our results showed that the concentrations of total PAEs in conventional, soil-cultivated vegetables and hydroponic vegetables are not significantly different. However, more PAE compounds appear in conventional vegetables (Figure 2). The PAEs in conventional vegetables mainly come from soil and the mulch film that is used in the planting process [29,30]. Irrigation water and the PAE-containing hydroponic facilities are the primary sources of PAEs in hydroponic vegetables [31]. We recommend using PAE-free water for irrigation and avoiding using PAE-containing facilities to reduce the PAEs residue in hydroponic vegetables.

### 3.3. Comparison of the Heavy Metals between Hydroponic and Conventional Vegetables

**Figure 3:**
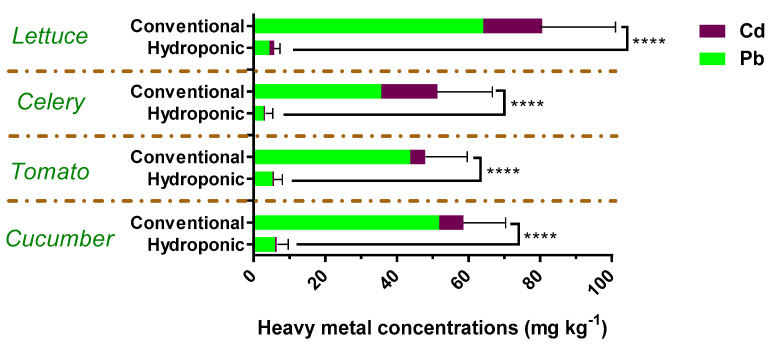
Heavy metal accumulation in vegetables from hydroponic and conventional soil-based cultivation. Values represent means ± SDs, **** *p* < 0.0001. Statistical significance was assessed with *t*-tests.

The lead and cadmium concentrations of all vegetable samples were determined in this study. Figure 3 shows that the levels of these two heavy metals were higher in conventional vegetables than in hydroponic vegetables. We found a significant difference in the total heavy metal concentrations between conventional and hydroponic vegetables (*p* < 0.0001). They were 16.0 and 14.1 times higher in lettuce and celery, 9.2 times higher in cucumber, and 8.6 times higher in tomatoes. Among the conventional vegetables, the lettuce had the highest average total heavy metal concentrations (all) of 80.6 μg kg^−1^, followed by cucumber with 58.6 μg kg^−1^, celery with 51.3 μg kg^−1^, and tomato with 47.9 μg kg^−1^. Among the hydroponic vegetables, cucumber contained the highest total heavy metal levels, with an average value of 6.4 μg kg^−1^, followed by lettuce (average value 5.7 μg kg^−1^), tomato (average value 5.6 μg kg^−1^), and celery (average value 3.2 μg kg^−1^). Lead was the leading heavy metal pollutant in all vegetable samples, with a 69–91% detection frequency. The detectable rates of lead and cadmium in the conventional lettuce samples were about twice as high as that in the hydroponic lettuce. The results for celery, tomato, and cucumber, on the other hand, demonstrated similar detection rates of lead and cadmium in conventional and hydroponic samples (SI Table S6). Soil and irrigation water are the primary sources of heavy metal residues in conventional and hydroponic vegetables, respectively. The accumulation of lead and cadmium in the vegetable planting soil in Shanghai were 13.02 ± 4.89 mg kg^−1^ and 0.13 ± 0.02 mg kg^−1^, respectively. The concentrations of lead and cadmium in the irrigation water of hydroponic vegetables in this study were 0.036 ± 0.012 mg L^−1^ and 0.00017 ± 0.00008 mg L^−1^, respectively. There was a large difference in the concentration of heavy metals in the growing environment of hydroponic and conventional soil-based vegetables. Additionally, hydroponic vegetables require less growing time, which avoids further heavy metal accumulation.

### 3.4. Risk Assessment of Vegetables from Hydroponic and Conventional Cultivation

The risk assessment results for the four vegetables produced from hydroponic and conventional cultivation are summarized in Table 2 and Table 3. Considering the overall dataset (n = 177), the average EDI of each pesticide was less than the acceptable daily intake (ADI) for adults in China. The average EDI of PAEs and heavy metals for adults from all the samples were less than the applied TDI and RfD that were obtained from the US EPA. 

THQ has been recognized as a significant parameter for assessing non-carcinogenic health risks associated with consuming food contaminated by toxic elements. The THQ results for pesticides, PAEs, and heavy metals in vegetables from hydroponic and conventional cultivation are listed in Table 2 and Table 3. If the THQ value is less than one, the exposed people are unlikely to be exposed to a toxic level with possible consequences for health. This study showed no THQ values exceeding one by consuming the above-studied vegetables in the local Shanghai area. The results revealed insignificant potential health risks associated with pesticides, PAEs, and heavy metals in vegetables from the Shanghai area.

Pests, weeds, and diseases pose a severe risk of primary producers impacting the market access and agricultural production. Most of the vegetable samples in this study contained more than one pesticide, because insecticide and fungicide mixtures are widely used to enhance their spectrum and efficacy for integrated pest management (IPM) purposes. In addition, PAEs and heavy metals were detected in nearly all the samples. The measurement of the potential adverse health effects to consumers from a mixture of pesticides, PAEs, and heavy metals in vegetables from hydroponic and conventional cultivation was evaluated by estimating the HI values. Conventionally, an HI value below one indicates that the total exposure does not exceed the considered acceptable level, and customers are unlikely to experience immediate toxic effects. This study’s average HI values in hydroponic and conventional vegetables were 0.22 and 0.64, respectively. Although both values were lower than 1, the difference between hydroponic and conventional vegetables is significant. The consumption of hydroponic vegetables poses fewer potential health risks than conventional soil-based vegetables.

In contrast to other studies, the HI in our study included pesticides, PAEs, and heavy metals. In hydroponic vegetables, pesticides were the key components contributing to the potential health risk (76.05%), followed by PAEs (20.06%) and heavy metals (3.89%). In the conventional soil-based vegetables, pesticides were still the dominant contaminants (77.37%), followed by heavy metals (14.96%) and PAEs (7.67%). The HIpesticides of the hydroponic and conventional vegetables were 0.17 and 0.50, respectively. Our results showed that hydroponic cultivation could avoid pests and disease in vegetables and reduce pesticides. However, hydroponic cultivation cannot reduce the health risk of PAEs. The HIPAEs of the hydroponic and conventional vegetables were 0.044 and 0.049, respectively. The HIheavy metal value for hydroponic vegetables (0.0085) was much lower than that of conventional soil-based vegetables (0.096). This difference demonstrated that hydroponic cultivation helps vegetables avoid heavy metal pollution.

Our study showed that the consumption of hydroponic vegetables causes a lower health risk to consumers. However, there is still a need to monitor the residues of pesticides and PAEs in hydroponic vegetables. Studies have shown that pesticides contaminate organic foods much less than they do conventional foods [6]. However, there are no significant differences in organic produce compared to conventional produce [32]. The hydroponic system avoids heavy metal pollution due to its soilless feature. Therefore, it is critical to use pollution-free water in hydroponic vegetable cultivation to avoid heavy metal pollution altogether. The integration of the hydroponic system with municipal wastewater treatment has the advantage of reducing costs in terms of pollutant removal, while reducing the maintenance and energy costs that come with conventional wastewater treatment [26]. There are not many studies comparing conventional soil-based and hydroponic products. Our results exhibit that hydroponic vegetables pose lower health risks than their conventional counterparts.

## 4. Conclusions

We studied the occurrence and risk assessment of various pesticides, PAEs, and heavy metals in four vegetables (lettuces, celeries, tomatoes, and cucumbers, n = 177) from hydroponic and conventional soil-based cultivation. Overall, at least one pesticide was detected in 53% of all the tested samples. While 84% of the conventionally grown samples contained at least one pesticide, 30% of the hydroponic samples carried detectable pesticide residues. The detection rate of pesticides in hydroponic vegetables was significantly lower than that in conventional vegetables. No significant difference was found between the conventional and hydroponic vegetables regarding the total PAE concentrations. More PAEs (e.g., DEP, DMEP, DMP, DHP, and DPP) were detected in conventional soil-based vegetables, while only three PAEs (DEHP, DnBP, and DiBP) were detected in the hydroponic vegetables. We found that the lead and cadmium residues in conventional vegetables were significantly higher than those in the hydroponic vegetables. In all vegetable samples, lead was the primary heavy metal pollutant. The hydroponic and conventional vegetables’ HI values were 0.22 and 0.64, respectively. Since both values were less than one, exposure to these hazardous chemicals through consumption of the studied vegetables may not pose significant health risks. It is suggested that the health risks of the consumption of hydroponic vegetables are lower compared to conventional soil-based vegetables. In the hydroponic vegetable samples, pesticides and PAEs were the key components contributing to the potential health risk. Using pollution-free water and monitoring pesticides in the cultivation of hydroponic vegetables may further reduce the health risk to consumers.

## Figures and Tables

**Table 1:** Commodity, variety, and farming method (sample number of hydroponic and conventional soil-based cultivation) of the samples collected in the present survey (*n* = 177).

Commodity	Variety	Hydroponic Farming (Sample Number, *n*)	Conventional Farming (Sample Number, *n*)
Lettuce	Romaine	18	11
	Iceberg	6	9
	Boston	8	6
Celery	Chinese celery	9	10
	American celery	6	10
Tomato	Roma	5	7
	Cherry	10	9
	Round	5	6
Cucumber	Holland cucumber	10	9
	Chinese cucumber	9	14

**Table 2:** Contaminant concentrations, EDI and THQ value, detection rate, and excess MRL rate of pesticide, PAE and heavy metal concentrations detected in hydroponic vegetables.

Contaminants	Mean Value (μg/kg)	Median Value (μg/kg)	Maximum Value (μg/kg)	EDI ^a^ (μg/kg·d)	THQ ^b^	Detection Rate (%)	Excess MRL Rate (%)
Acetamiprid	3.28 × 10	1.10 × 10	1.20 × 10^2^	1.33 × 10^−1^	1.89 × 10^−3^	5.81	0
Boscalid	9.00	9.00	9.00	3.63 × 10^−2^	9.08 × 10^−4^	1.16	0
Carbendazim	2.60 × 10	1.80 × 10	5.00 × 10	1.05 × 10^−1^	3.50 × 10^−3^	1.16	0
Chlorantraniliprole	7.40 × 10	2.00 × 10	2.47 × 10^2^	2.98 × 10^−1^	1.49 × 10^−4^	4.65	0
Chlorfenapyr	8.08 × 10	8.08 × 10	1.38 × 10^2^	3.26 × 10^−1^	1.09 × 10^−2^	2.33	0
Chlorpyrifos	1.30 × 10	1.30 × 10	1.30 × 10	5.24 × 10^−2^	5.24 × 10^−3^	2.33	0
Cyhalothrin	1.80 × 10	1.80 × 10	1.80 × 10	7.26 × 10^−2^	3.63 × 10^−3^	1.16	0
Cymoxanil	1.40 × 10	1.40 × 10	1.40 × 10	5.65 × 10^−2^	4.34 × 10^−3^	1.16	0
Cypermethrin	5.82 × 10	5.82 × 10	5.82 × 10	2.35 × 10^−1^	1.17 × 10^−2^	1.16	0
Cyromazine	1.50 × 10	1.50 × 10	1.50 × 10	6.05 × 10^−2^	1.01 × 10^−3^	1.16	0
Dimethomorph	1.45 × 10^2^	1.45 × 10^2^	1.45 × 10^2^	5.85 × 10^−1^	2.92 × 10^−3^	2.33	0
Indoxacard	2.02 × 10^2^	2.02 × 10^2^	2.02 × 10^2^	8.15 × 10^−1^	8.15 × 10^−2^	1.16	0
Lufenuron	7.48 × 10	7.48 × 10	7.48 × 10	3.02 × 10^−1^	2.01 × 10^−2^	1.16	0
Metalaxyl	4.00 × 10	4.00 × 10	4.00 × 10	1.61 × 10^−1^	2.02 × 10^−3^	1.16	0
Nitenpyram	9.00	9.00	9.00	3.63 × 10^−2^	6.85 × 10^−5^	2.33	0
Procymidone	3.70 × 10^2^	3.70 × 10^2^	3.70 × 10^2^	1.49	1.49 × 10^−2^	1.16	0
Thiamethoxam	2.00 × 10	2.00 × 10	2.00 × 10	8.07 × 10^−2^	1.01 × 10^−3^	2.33	0
DEHP	1.41 × 10^3^	9.70 × 10^2^	6.72 × 10^3^	5.69	2.84 × 10^−2^	84.88	—
DiBP	1.21 × 10^3^	8.50 × 10^2^	4.00 × 10^3^	4.88	9.76 × 10^−3^	86.05	—
DnBP	8.00 × 10^2^	7.70 × 10^2^	2.73 × 10^3^	3.23	5.55 × 10^−3^	83.72	—
Pb	4.77	3.60	1.34 × 10	1.92 × 10^−2^	4.81 × 10^−3^	68.60	0
Cd	9.10 × 10^−1^	6.50 × 10^−1^	5.10	3.67 × 10^−3^	3.67 × 10^−3^	46.51	0

^a^ EDI, estimated daily intake. ^b^ THQ, target hazard quotient.

**Table 3:** Contaminant concentrations, EDI and THQ value, detection rate, and excess MRL rate of pesticide, PAE, and heavy metal concentrations detected in conventional soil-based vegetables.

Contaminants	Mean Value (μg/kg)	Median Value (μg/kg)	Maximum Value (μg/kg)	EDI ^a^ (μg/kg·d)	THQ ^b^	Detection Rate (%)	Excess MRL Rate (%)
Abamectin	1.20 × 10	1.20 × 10	1.40 × 10	4.84 × 10^−2^	2.42 × 10^−2^	2.20	0
Acetamiprid	2.74 × 10^2^	7.25 × 10^2^	1.75 × 10^3^	1.11	1.58 × 10^−2^	31.87	3.30
Azoxystrobin	2.10 × 10	2.10 × 10	2.10 × 10	8.47 × 10^−2^	4.24 × 10^−4^	3.30	0
Bifenthrin	1.05 × 10	1.05 × 10	1.05 × 10	4.24 × 10^−2^	4.24 × 10^−3^	2.20	0
Boscalid	1.12 × 10^2^	2.00 × 10	5.80 × 10^2^	4.52 × 10^−1^	1.13 × 10^−2^	7.69	1.10
Carbendazim	6.98 × 10	5.20 × 10	1.60 × 10^2^	2.82 × 10^−1^	9.38 × 10^−3^	10.99	0
Chlorantraniliprole	1.70 × 10^2^	7.45 × 10	5.43 × 10^2^	6.86 × 10^−1^	3.43 × 10^−4^	15.38	0
Chlorfenapyr	1.90 × 10^2^	1.18 × 10^2^	5.29 × 10^2^	7.66 × 10^−1^	2.55 × 10^−2^	10.99	1.10
Chlorpyrifos	1.30 × 10	1.30 × 10	1.50 × 10	5.24 × 10^−2^	5.24 × 10^−3^	2.20	0
Cyhalothrin	1.10 × 10^2^	1.10 × 10^2^	1.10 × 10^2^	4.44 × 10^−1^	2.22 × 10^−2^	1.10	0
Cymoxanil	1.85 × 10	1.23 × 10	3.30 × 10	7.46 × 10^−2^	5.74 × 10^−3^	5.49	0
Cypermethrin	5.78 × 10	5.78 × 10	5.82 × 10	2.33 × 10^−1^	1.17 × 10^−2^	2.20	0
Cyromazine	4.80 × 10^2^	4.00 × 10	3.35 × 10^3^	1.94	3.23 × 10^−2^	9.89	1.10
Difenoconazole	1.23 × 10^2^	6.70 × 10	3.72 × 10^2^	4.96 × 10^−1^	4.96 × 10^−2^	16.48	0
Dimethomorph	1.67 × 10^2^	1.42 × 10^2^	4.10 × 10^2^	6.74 × 10^−1^	3.37 × 10^−3^	15.38	0
Flusilazole	1.54 × 10^2^	1.54 × 10^2^	1.57 × 10^2^	6.21 × 10^−1^	8.87 × 10^−2^	2.20	0
Imidacloprid	1.32 × 10^2^	2.90 × 10	6.43 × 10^2^	5.32 × 10^−1^	8.87 × 10^−3^	12.09	0
Indoxacard	2.58 × 10^2^	2.58 × 10^2^	3.01 × 10^2^	1.04	1.04 × 10^−1^	2.20	0
Lufenuron	1.18 × 10^2^	7.03 × 10	3.30 × 10^2^	4.76 × 10^−1^	3.17 × 10^−2^	5.49	0
Metalaxyl	6.94 × 10	6.82 × 10	7.20 × 10	2.80 × 10^−1^	3.45 × 10^−3^	3.30	0
Myclobutanil	2.53 × 10	2.53 × 10	2.56 × 10	1.02 × 10^−1^	3.40 × 10^−3^	2.20	0
Paclobutrazol	1.15 × 10	1.15 × 10	1.20 × 10	4.64 × 10^−2^	4.64 × 10^−4^	2.20	0
Pendimethalin	1.08 × 10	1.08 × 10	1.10 × 10	4.36 × 10^−2^	1.45 × 10^−3^	2.20	0
Procymidone	1.09 × 10	1.09 × 10	1.09 × 10	4.40 × 10^−2^	4.40 × 10^−4^	1.10	0
Propamocarb	2.14 × 10^2^	2.14 × 10^2^	2.20 × 10^2^	8.63 × 10^−1^	2.16 × 10^−3^	2.20	0
Propiconazole	3.67 × 10	8.70	9.31 × 10	1.48 × 10^−1^	2.12 × 10^−3^	3.30	0
Pyraclostrobin	5.58 × 10	2.01 × 10	1.27 × 10^2^	2.25 × 10^−1^	7.50 × 10^−3^	3.30	0
Pyridaben	4.53 × 10	2.60 × 10	1.18 × 10^2^	1.83 × 10^−1^	1.82 × 10^−2^	7.69	0
Thiamethoxam	4.10 × 10	3.40 × 10	5.90 × 10	1.65 × 10^−1^	2.07 × 10^−3^	5.49	0
DEHP	1.29 × 10^3^	9.75 × 10^2^	5.33 × 10^3^	5.20	2.60 × 10^−2^	74.73	—
DEP	7.45 × 10	5.00 × 10	2.84 × 10^2^	3.00 × 10^−1^	6.01 × 10^−4^	69.23	—
DiBP	1.38 × 10^3^	7.15 × 10^2^	5.71 × 10^3^	5.57	1.11 × 10^−2^	74.73	—
DMEP	4.70 × 10^2^	3.90 × 10^2^	1.67 × 10^3^	1.90	3.79 × 10^−3^	28.57	—
DMP	8.78	7.26	4.47 × 10	3.54 × 10^−2^	7.08 × 10^−5^	39.56	—
DnBP	9.36 × 10^2^	9.30 × 10^2^	2.80 × 10^3^	3.78	7.55 × 10^−3^	74.73	—
DPP	4.54	1.00	2.00 × 10	1.83 × 10^−2^	3.66 × 10^−5^	13.19	—
Pb	4.91 × 10	3.64 × 10	2.77 × 10^2^	1.98 × 10^−1^	4.95 × 10^−2^	79.12	1.10
Cd	1.15 × 10	8.48	3.30 × 10	4.64 × 10^−2^	4.64 × 10^−2^	81.32	0

^a^ EDI, estimated daily intake. ^b^ THQ, target hazard quotient.

## Data Availability

The original contributions presented in the study are included in the article, further inquiries can be directed to the corresponding author.

## Supplementary Materials

The following supporting information can be downloaded at https://www.mdpi.com/article/10.3390/foods13081151/s1, Table S1: The 120 pesticides measured in this study; Table S2: The 18 phthalates measured in this study; Table S3: Retention time (RT) and MRM condition of pesticides for UPLC-MS/MS analysis; Table S4: Retention times and MRM parameters of pesticides for GC-MS/MS; Table S5: Retention times and MRM parameters of phthalates for GC-MS/MS; Table S6: The detection rates of Pb and Cd in conventionally and hydroponically produced vegetable samples.
