# Supplementary material for: Occurrence and Risk Assessment of Pesticides, Phthalates, and Heavy Metal Residues in Vegetables from Hydroponic and Conventional Cultivation
Source: Foods. 2024 Apr 10;13(8):1151. doi: 10.3390/foods13081151 (PMC11049364; doi:10.3390/foods13081151)
Supplement: Supplementary file 1 [file foods-13-01151-s001.zip › foods-2782158-supplementary.pdf]

SUPPORTING MATERIALS

Table S1. 120 pesticides in this study

Pesticide	Molecular formula	Molecular weight	CAS number
Abamectin	$C_{48}H_{72}O_{14}$	873.09	65195-55 -3
Acephate	$C_4H_{10}NO_3PS$	183.17	30560-19-1
Acetamiprid	$C_{10}H_{11}ClN_4$	222.68	135410-20-7
Acetochlor	$C_{14}H_{20}ClNO_2$	269.8	34256-82-1
Acrinathrin	$C_{26}H_{21}F_6NO_5$	541.44	101007-06-1
Alachlor	$C_{14}H_{20}ClNO_2$	269.77	15972-60-8
Azoxystrobin	$C_{22}H_{17}N_3O_5$	403.39	131860-33-8
Bifenthrin	$C_{23}H_{22}ClF_3O_2$	422.87	82657-04-3
Boscalid	$C_{18}H_{12}Cl_2N_2O$	343.21	188425-85-6
Bromopropylate	$C_{17}H_{16}Br_2O_3$	428.11	18181-80-1
Buprofezin	$C_{16}H_{23}N_3OS$	305.4	69327-76-0
Carbaryl	$C_{12}H_{11}NO_2$	201.22	63-25-2
Carbendazim	$C_9H_9N_3O_2$	191.2	10605-21-7
Carbofuran	$C_{12}H_{15}NO_3$	221.25	1563-66-2
Carbophenothion	$C_{11}H_{16}ClO_2PS_3$	342.87	786-19-6
Carboxin	$C_{12}H_{13}NO_2S$	237.29	5234-68-4
Chloantraniliprole	$C_{18}H_{14}BrCl_2N_5O_2$	483.15	500008-45-7
Chlorbenzuron	$C_{14}H_{10}Cl_2N_2O_2$	309.14	196791-54-5
Chlorfenapyr	$C_{15}H_{11}BrClF_3N_2O$	407.62	122453-73-0
Chlorfenson	$C_{12}H_8Cl_2O_3S$	303.16	80-33-1
Chlorfluazuron	$C_{20}H_9C_{13}F_5N_3O_3$	540.65	71422-67-8
Chlormequat	$C_5H_{13}Cl_2N$	158.07	999-81-5
Chlorothalonil	$C_8Cl_4N_2$	265.91	1897-45-6
Chlorpyrifos	$C_9H_{11}Cl_3NO_3PS$	350.59	220-864-4
Chlorpyrifos-methyl	$C_7H_7Cl_3NO_3PS$	322.53	5598-13-0
Chlorthiophos	$C_{11}H_{15}Cl_2O_3PS_2$	361.24	60238-56-4
Clofentezine	$C_{14}H_8Cl_2N_4$	303.15	74115-24-5
Clothianidin	$C_6H_8ClN_5O_2S$	249.7	210880-92-5
Cyflufenamid	$C_{20}H_{17}F_5N_2O_2$	412.4	180409–60–3
Cyfluthrin	$C_{22}H_{18}Cl_2FNO_3$	434.29	68359-37-5
Cyhalothrin	$C_{23}H_{19}ClF_3NO_3$	449.85	91465-08-6
Cymoxanil	$C_7H_{10}N_4O_3$	198.18	57966-95-7
Cypermethrin	$C_{22}H_{19}Cl_2NO_3$	416.30	52315-07-8
Cyproconazole	$C_{15}H_{18}ClN_3O$	291.78	94361-06-5
Cyprodinil	$C_{14}H_{15}N_3$	225.29	121552-61-2
Cyromazine	$C_6H_{10}N_6$	166.18	66215-27-8
Deltamethrin	$C_{22}H_{19}Br_2NO_3$	505.2	52918-63-5
Diazinon	$C_{12}H_{21}N_2O_3PS$	304.35	333-41-5
Dichlorvos	$C_4H_7Cl_2O_4P$	220.98	62-73-7

Dicofol	$C_{14}H_9Cl_5O$	370.49	115-32-2
Diethofencarb	$C_{14}H_{21}NO_4$	267.32	87130-20-9
Difenoconazole	$C_{19}H_{17}Cl_2N_3O_3$	406.27	119446-68-3
Diflubenzuron	$C_{14}H_9ClF_2N_2O_2$	310.68	35367-38-5
Dimethoate	$C_5H_{12}NO_3PS_2$	229.26	60-51-5
Dimethomorph	$C_{21}H_{22}ClNO_4$	387.86	110488-70-5
Emamectin benzoate	$C_{49}H_{77}NO_{13}$	888.13	155569-91-8
Fenhexamid	$C_{14}H_{17}Cl_2NO_2$	302.20	126833-17-8
Fenitrothion	$C_9H_{12}NO_5PS$	277.23	122-14-5
Fenobucarb	$C_{12}H_{17}NO_2$	207.27	3766-81-2
Fenothiocarb	$C_{13}H_{19}NO_2S$	253.36	62850-32-2
Fenpropathrin	$C_{22}H_{23}NO_3$	349.42	39515-41-8
Fenthion	$C_{10}H_{15}O_3PS_2$	278.20	55-38-9
Fenvalerate	$C_{25}H_{22}ClNO_3$	419.9	51630-58-1
Fipronil	$C_{12}H_4Cl_2F_6N_4OS$	437.15	120068-37-3
Fluazinam	$C_{13}H_4Cl_2F_6N_4O_4$	465.09	79622-59-6
Flucythrinate	$C_{26}H_{23}F_2NO_4$	451.47	70124-77-5
Flusilazole	$C_{16}H_{15}F_2N_3Si$	315.4	85509-19-9
Fosthiazate	$C_9H_{18}NO_3PS_2$	283.35	98886-44-3
Hexaconazole	$C_{14}H_{17}Cl_2N_3O$	314.21	79983-71-4
Imidacloprid	$C_9H_{10}ClN_5O_2$	255.66	138261-41-3
Indoxacarb	$C_{22}H_{17}ClF_3N_3O_7$	527.83	144171-61-9
Iprodione	$C_{13}H_{13}Cl_2N_3O_3$	330.17	36734-19-7
Isazofos	$C_9H_{17}ClN_3O_3PS$	313.74	42509-80-8
Isocarbophos	$C_{11}H_{16}NO_4PS$	289.29	24353-61-5
Isofenphos-methyl	$C_{14}H_{22}NO_4PS$	331.37	99675-03-3
Isoprocarb	$C_{11}H_{15}NO_2$	193.24	2631-40-5
Isoxathion	$C_{13}H_{16}NO_4PS$	313.31	18854-01-8
Kresoxim-methyl	$C_{18}H_{19}NO_4$	313.35	143390-89-0
Lufenuron	$C_{17}H_8Cl_2F_8N_2O_3$	511.15	103055-07-8
Malaoxon	$C_{10}H_{19}O_7PS$	314.29	1634-78-2
Malathion	$C_{10}H_{19}O_6PS_2$	330.36	121-75-5
Mepanipyrim	$C_{14}H_{13}N_3$	223.27	110235-47-7
Mepiquat chloride	$C_7H_{16}ClN$	149.66	24307-26-4
Metalaxyl	$C_{15}H_{21}NO_4$	279.33	57837-19-1
Methacrifos	$C_7H_{13}O_5PS$	240.21	62610-77-9
Methamidophos	$C_2H_8NO_2PS$	141.13	10265-92-6
Methomyl	$C_5H_{10}N_2O_2S$	162.21	16752-77-5
Monocrotophos	$C_7H_{14}NO_5P$	223.16	6923-22-4
Myclobutanil	$C_{15}H_{17}ClN_4$	288.78	88671-89-0
Naled	$C_4H_7Br_2Cl_2O_4P$	380.78	300-76-5
Napropamide	$C_{17}H_{21}NO_2$	271.35	15299-99-7
Omethoate	$C_5H_{12}NO_4PS$	213.19	1113-02-6

Paclobutrazol	$C_{15}H_{20}ClN_3O$	293.8	76738-62-0
Parathion	$C_{10}H_{14}NO_5PS$	291.26	56-38-2
Parathion-methyl	$C_8H_{10}NO_5PS$	263.21	298-00-0
Penconazole	$C_{13}H_{15}Cl_2N_3$	284.18	66246-88-6
Pendimethalin	$C_{13}H_{19}N_3O_4$	281.31	40487-42-1
Phorate	$C_7H_{17}O_2PS_3$	260.38	298-02-2
Phorate sulfone	$C_7H_{17}O_4PS_3$	292.38	2588-04-7
Phorate sulfoxide	$C_7H_{17}O_3PS_3$	276.38	2588-03-6
Phosalone	$C_{12}H_{15}ClNO_4PS_2$	367.81	2310-17-0
Phosmet	$C_{11}H_{12}NO_4PS_2$	317.32	732-11-6
Phoxim	$C_{12}H_{15}N_2O_3PS$	298.30	14816-18-3
Prochloraz	$C_{15}H_{16}Cl_3N_3O_2$	376.7	67747-09-5
Procymidone	$C_{13}H_{11}Cl_2NO_2$	284.14	32809-16-8
Profenofos	$C_{11}H_{15}BrClO_3PS$	373.63	41198-08-7
Propamocarb	$C_9H_{20}N_2O_2$	188.27	24579-73-5
Propiconazole	$C_{15}H_{17}C_{12}N_3O_2$	342.22	60207-90-1
Pyraclostrobin	$C_{19}H_{18}N_3O_4Cl$	387.82	175013-18-0
Pyridaben	$C_{19}H_{25}ClN_2OS$	364.93	96489-71-3
Pyrimethanil	$C_{12}H_{13}N_3$	199.25	53112-28-0
Quinalphos	$C_{12}H_{15}N_2O_3PS$	298.3	13593-03-8
Quinoxyfen	$C_{15}H_8Cl_2FNO$	308.13	124495-18-7
Spirodiclofen	$C_{21}H_{24}Cl_2O_4$	411.32	148477-71-8
Sulprofos	$C_{12}H_{19}O_2PS_3$	322.45	35400-43-2
Tebuconazole	$C_{16}H_{22}ClN_3O$	307.82	107534-96-3
Tebufenozide	$C_{22}H_{28}N_2O_2$	352.47	112410-23-8
Tecnazene	$C_6HCl_4NO_2$	260.89	117-18-0
Terbufos	$C_9H_{21}O_2PS_3$	288.43	13071-79-9
Terbufos sulfone	$C_9H_{21}O_4PS_3$	320.43	56070-16-7
Tetramethrin	$C_{19}H_{25}NO_4$	331.41	7696-12-0
Thiamethoxam	$C_8H_{10}ClN_5O_3S$	291.71	153719-23-4
Thiobencarb	$C_{12}H_{16}ClNOS$	257.78	28249-77-6
Thiophanate-methyl	$C_{12}H_{14}N_4O_4S_2$	342.39	23564-05-8
Triadimefon	$C_{14}H_{16}ClN_3O_2$	293.75	43121-43-3
Triazophos	$C_{12}H_{16}N_3O_3PS$	313.31	24017-47-8
Tridemorph	$C_{19}H_{39}NO$	297.52	24602-86-6
Uniconazole	$C_{15}H_{18}ClN_3O$	291.78	83657-17-4
Vamidothion	$C_8H_{18}NO_4PS_2$	287.34	2275-23-2
Vinclozolin	$C_{12}H_9Cl_2NO_3$	286.11	50471-44-8

Table S2. 18 phthalates in this study

Phthalate	Molecular formula	Molecular weight	CAS number
Benzyl butyl phthalate	$C_{19}H_{20}O_4$	312.36	85-68-7
Bis(2-butoxyethyl)	$C_{20}H_{30}O_6$	366.45	117-83-9

phthalate			
Bis(2-ethoxyethyl) Phthalate	$C_{16}H_{22}O_6$	310.34	605-54-9
Bis(2-ethylhexyl) phthalate	$C_{24}H_{38}O_4$	390.56	117-81-7
Bis(2-methoxyethyl) phthalate	$C_{14}H_{18}O_6$	282.29	117-82-8
Bis(4-methyl-2-pentyl) phthalate	$C_{20}H_{30}O_4$	334.45	146-50-9
Dibenzyl phthalate	$C_{22}H_{18}O_4$	346.38	523-31-9
Dibutyl phthalate	$C_{16}H_{22}O_4$	278.34	84-74-2
Dicyclohexyl phthalate	$C_{20}H_{26}O_4$	330.42	84-61-7
Diethyl phthalate	$C_{12}H_{14}O_4$	222.24	84-66-2
Dihexyl phthalate	$C_{20}H_{30}O_4$	334.45	84-75-3
Diisobutyl phthalate	$C_{16}H_{22}O_4$	278.34	84-69-5
Diisodecyl phthalate	$C_{28}H_{46}O_4$	446.66	26761-40-0
Diisononyl phthalate	$C_{26}H_{42}O_4$	418.61	28553-12-0
Dimethyl phthalate	$C_{10}H_{10}O_4$	194.18	131-11-3
Dinoctyl phthalate	$C_{24}H_{38}O_4$	390.56	117-84-0
Dinonyl phthalate	$C_{26}H_{42}O_4$	418.61	84-76-4
Dipentyl phthalate	$C_{18}H_{26}O_4$	306.4	131-18-0

Table S3. Retention time (RT) and MRM condition of pesticides for UPLC-MS/MS analysis.

Analyte	**t_R[a] (min)**	**Precursor ion (m/z)**	**Product ion (m/z)**	**DP[b] (V)**	**CE[c] (eV)**
Abamectin 1	23.44	895.5	751.4	195	57
Abamectin 2	23.44	895.5	449.2	195	64
Acephate1	2.1	184	143	50	10
Acephate2	2.1	184	125	50	26
Acetamiprid 1	3.7	223	126	70	27
Acetamiprid 2	3.7	223	99	70	47
Acetochlor 1	7.9	270.2	148.2	36	15
Acetochlor 2	7.9	270.2	133.1	36	45
Alachlor 1	7.9	270.1	238.1	46	15
Alachlor 2	7.9	270.1	162	46	25
Azoxystrobin 1	6.7	404.1	372	70	20
Azoxystrobin 2	6.7	404.1	344.1	70	34
Buprofezin 1	9.5	306.2	201.1	66	17
Buprofezin 2	9.5	306.2	116.2	66	21
Carbaryl 1	5.3	202.1	145	54	15
Carbaryl 2	5.3	202.1	127	54	40
Carbendazim 1	4.1	192	160	80	25

Carbendazim 2	4.1	192	132	80	41
Carbofuran 1	5.0	222.1	165	70	17
Carbofuran 2	5.0	222.1	123.1	70	29
Carboxin 1	5.4	236.1	142.9	70	21
Carboxin 2	5.4	236.1	87	70	33
Chlorantraniliprole 1	6.5	483.9	452.9	80	28
Chlorantraniliprole 2	6.5	483.9	285.9	80	28
Chlorbenzuron 1	8.3	309	156	75	20
Chlorbenzuron 2	8.3	309	139	75	44
Chlorfluazuron 1	10.4	540	383	110	30
Chlorfluazuron 2	10.4	540	158	110	27
Chlormequat 1	1.5	121.9	57.7	38	20
Chlormequat 2	1.5	121.9	62.4	38	20
Chlorpyrifos 1	10.0	350	198	82	29
Chlorpyrifos 2	10.0	350	97	82	49
Chlorpyrifos-methyl 1	9.0	324	125.1	65	28
Chlorpyrifos-methyl 2	9.0	321.9	125.1	65	25
Clofentezine 1	17.87	303.0	138.0	51	21
Clofentezine 2	17.87	303.0	102.0	51	47
Clothianidin 1	3.5	250	169.1	71	17
Clothianidin 2	3.5	250	132	71	21
Cymoxanil 1	8.56	199.1	128.1	45	12
Cymoxanil 2	8.56	199.1	111.1	45	25
Cyromazine 1	1.01	167.5	125.0	60	24
Cyromazine 2	1.01	167.5	85.0	60	24
Deltamethrin 1	10.5	523.2	281	55	23
Deltamethrin 2	10.5	523.2	506.1	55	16
Diazinon 1	8.6	305	169	80	27
Diazinon 2	8.6	305	153	80	28
Dichlorvos 1	4.9	221	109	70	23
Dichlorvos 2	4.9	221	127	70	25
Diethofencarb 1	6.9	268.1	226.1	66	14
Diethofencarb 2	6.9	268.1	180	66	25
Difenoconazole 1	8.9	406.1	251	120	37
Difenoconazole 2	8.9	406.1	337	120	23
Diflubenzuron 1	8.0	311	158	72	21
Diflubenzuron 2	8.0	311	141.2	72	47
Dimethoate 1	3.7	230	125	56	29
Dimethoate 2	3.7	230	199	56	13
Dimethomorph 1	7.1	388.1	301	115	29
Dimethomorph 2	7.1	388.1	165	115	43
Emamectin benzoate 1	21.62	886.5	82.1	50	110
Emamectin benzoate 2	21.62	886.5	158.1	50	41

Fenobucarb 1	6.8	208.1	95	65	21
Fenobucarb 2	6.8	208.1	152	65	13
Fenothiocarb 1	8.2	254.1	72.1	61	35
Fenothiocarb 2	8.2	254.1	160.2	61	14
Fenthion 1	8.4	279.1	169	78	23
Fenthion 2	8.4	279.1	247	78	18
Fipronil 1	8.1	454	368.1	50	33
Fipronil 2	8.1	454	290.1	50	42
Fluazinam 1	21.55	462.9	415.9	-20	-31
Fluazinam 2	21.55	462.9	398.0	-20	-23
Flusilazole 1	15.14	316.1	247.1	50	26
Flusilazole 2	15.14	316.1	165.1	50	37
Fosthiazate 1	5.7	284	104	63	28
Fosthiazate 2	5.7	284	228	63	15
Hexaconazole 1	8.6	314.1	70.1	94	45
Hexaconazole 2	8.6	314.1	159	94	40
Imidacloprid 1	3.4	256.1	175	60	26
Imidacloprid 2	3.4	256.1	209	60	23
Indoxacarb 1	9.0	528.1	203	71	51
Indoxacarb 2	9.0	528.1	56	71	55
Iprodione 1	8.0	330.1	245	85	21
Iprodione 2	8.0	330.1	288	85	16
Isazofos 1	7.5	314	162	70	22
Isazofos 2	7.5	314	120	70	40
Isoxathion 1	8.7	314.1	105	59	21
Isoxathion 2	8.7	314.1	170	59	19
Lufenuron 1	16.94	508.9	489	37	11
Lufenuron 2	16.94	508.9	338.9	37	14
Malathion 1	7.3	331	127	64	17
Malathion 2	7.3	331	99	64	31
Mepiquat chloride	1.6	113.9	97.8	38	20
Metalaxyl 1	6.2	280.2	220	65	18
Metalaxyl 2	6.2	280.2	192.3	65	24
Methamidophos 1	1.7	142	125	54	18
Methamidophos 2	1.7	142	94	54	19
Methomyl 1	2.9	163	106	38	13
Methomyl 2	2.9	163	88	38	13
Monocrotophos 1	3.1	224.1	127	71	21
Monocrotophos 2	3.1	224.1	98	71	17
Myclobutanil 1	7.4	289.1	70	80	35
Myclobutanil 2	7.4	289.1	125	80	46
Napropamide 1	7.9	272	129.3	80	21
Napropamide 2	7.9	272	171	80	26

Omethoate 1	2.3	214	109	56	36
Omethoate 2	2.3	214	182.9	56	16
Paclobutrazol 1	7.2	294	70	90	50
Paclobutrazol 2	7.2	294	125	90	55
Parathion 1	8.2	292	236	80	20
Parathion 2	8.2	292	264	80	15
Penconazole 1	8.3	284	159	81	39
Penconazole 2	8.3	284	70	81	29
Pendimethalin 1	10.1	282.1	212	45	15
Pendimethalin 2	10.1	282.1	194	45	25
Phorate 1	8.8	261	75	51	21
Phorate 2	8.8	261	199	51	10
Phorate sulfone 1	5.9	293	96.9	65	50
Phorate sulfone 2	5.9	293	114.7	65	35
Phorate sulfoxide 1	5.7	276.9	96.9	60	45
Phorate sulfoxide 2	5.7	276.9	114.7	55	28
Phosalone 1	8.8	368	182	71	20
Phosalone 2	8.8	368	322	71	13
Phosmet 1	6.6	318	160	61	17
Phosmet 2	6.6	318	133	61	49
Phoxim 1	8.7	299.1	129	67	16
Phoxim 2	8.7	299.1	77	67	46
Prochloraz 1	8.8	376.2	308	65	17
Prochloraz 2	8.8	376.2	70.1	65	43
Profenofos 1	9.5	373	302.9	80	25
Profenofos 2	9.5	373	345.2	80	18
Propamocarb 1	1.06	189	102	80	15
Propamocarb 2	1.06	189	74	80	20
Propiconazole 1	8.5	342.1	159	86	43
Propiconazole 2	8.5	342.1	69.1	86	33
Pyraclostrobin 1	18.23	388.1	194.1	50	18
Pyraclostrobin 2	18.23	388.1	163.1	50	36
Pyrimethanil 1	7.0	200	107	91	34
Pyrimethanil 2	7.0	200	82	91	37
Quinalphos 1	8.3	299	163	66	31
Quinalphos 2	8.3	299	147	66	29
Quinoxyfen 1	10.0	308	162	61	57
Quinoxyfen 2	10.0	308	197	61	43
Spirodiclofen 1	10.6	411.2	71.1	46	25
Spirodiclofen 2	10.6	411.2	313.1	46	17
Sulprofos 1	10.1	323	219	81	21
Sulprofos 2	10.1	323	247	81	17
Tebuconazole 1	8.4	308.1	70	90	49

Tebuconazole 2	8.4	308.1	125	90	47
Tebufenozide 1	15.88	353.2	133.1	35	24
Tebufenozide 2	15.88	353.2	297.2	35	11
Thiamethoxam 1	3.0	292	211	60	18
Thiamethoxam 2	3.0	292	181	60	32
Thiobencarb 1	8.9	258.1	125	66	25
Thiobencarb 2	8.9	258.1	89	66	67
Thiophanate-methyl 1	5.62	343.1	151.0	60	26
Thiophanate-methyl 2	5.62	343.1	311.0	60	15
Triadimefon 1	7.4	294	197	81	21
Triadimefon 2	7.4	294	225	81	17
Triazophos 1	7.5	314	119.1	70	47
Triazophos 2	7.5	314	162	70	25
Tridemorph 1	17.6	298	130	50	25
Tridemorph 2	17.6	298	98	50	30
Uniconazole 1	8.0	292.1	124.9	90	40
Uniconazole 2	8.0	292.1	70	90	55
Vamidothion 1	3.7	288	146	56	17
Vamidothion 2	3.7	288	118	56	31

[a] Retention time; [b] Declustering potential; [c] Collision energy.

Table S4. Retention times and MRM parameters of pesticides for GC-MS/MS.

Pesticides	t_R(min)	MRM1	CE1 (ev)	MRM2	CE2 (ev)
Acrinathrin	31.86	288.9 -> 92.8	10	207.8 -> 181.1	10
Bifenthrin	29.79	181.2 -> 165.2	25	181.2 -> 166.2	10
Boscalid	34.77	140.0 -> 112.0	10	140.0 -> 76.0	25
Bromopropylate	30.50	185.0 -> 157.0	15	183.0 -> 155.0	15
Carbophenothion	29.01	153.0 -> 96.9	10	199.0 -> 143.0	10
Chlorfenapyr	25.89	408 -> 59	15	247 -> 227	15
Chlorfenson	27.32	175.0 -> 111.0	10	111.0 -> 75.0	15
Chlorothalonil	21.43	263.8 > 168	25	263.8 > 229	20
Chlorthiophos-1	27.80	324.8 -> 268.9	10	296.8 -> 268.9	5
Chlorthiophos-2	28.27	324.8 -> 268.9	10	296.8 -> 268.9	5
Cyflufenamid	27.67	118.1 -> 90.0	10	118.1 -> 89.0	25

Cypermethrin-1	33.85	163.0 -> 91.0	10	163.0 -> 127.0	5
Cypermethrin-2	34.13	163.0 -> 91.0	10	163.0 -> 127.0	5
Cypermethrin-3	34.19	163.0 -> 91.0	10	163.0 -> 127.0	5
Cypermethrin-4	34.32	163.0 -> 91.0	10	163.0 -> 127.0	5
Cyfluthrin-1	33.65	226.0 -> 206.0	15	198.9 -> 170.1	25
Cyfluthrin-2	33.89	226.0 -> 206.0	15	198.9 -> 170.1	25
Cyfluthrin-3	33.95	226.0 -> 206.0	15	198.9 -> 170.1	25
Cyfluthrin-4	34.09	226.0 -> 206.0	15	198.9 -> 170.1	25
lambda-Cyhalothrin-1	31.70	197.0 -> 141.0	10	197.0 -> 161.0	5
lambda-Cyhalothrin-2	31.97	197.0 -> 141.0	10	197.0 -> 161.0	5
Cyproconazole-1	29.09	139.0 -> 111.0	15	222.0 -> 125.1	15
Cyproconazole-2	29.10	139.0 -> 111.0	15	222.0 -> 125.1	15
Cyprodinil	23.83	225.2 -> 224.3	10	224.2 -> 208.2	20
o,p'-Dicofol	23.29	139 > 111	15	250.9 > 138.9	15
Fenhexamid	26.17	177.1 -> 78.0	10	97.1 -> 55.1	10
Fenitrothion	23.67	277.0 -> 260.0	5	277.0 -> 109.0	20
Fenpropathrin	30.75	207.9 -> 181.0	5	264.9 -> 210.0	10
Fenvalerate-1	35.32	167.0 -> 125.1	5	224.9 -> 119.0	15
Fenvalerate-2	35.76	167.0 -> 125.1	5	224.9 -> 119.0	15
Flucythrinate-1	34.45	156.9 -> 107.1	15	198.9 -> 157.0	10
Flucythrinate-2	34.80	156.9 -> 107.1	15	198.9 -> 157.0	10
Isocarbophos	25.09	135.9 -> 108.0	15	135.9 -> 69.0	30

Isofenphos-methyl	24.47	199.0 -> 121.0	10	241.1 -> 199.1	10
Isoprocarb	15.07	121.0 -> 77.1	20	136.0 -> 121.1	10
Kresoxim-methyl	26.94	116.0 -> 89.0	15	116.0 -> 63.0	30
Malaoxon	22.60	126.9 -> 99.0	5	126.9 -> 55.0	5
Mepanipyrim	26.11	223.2 -> 222.2	10	222.2 -> 207.2	15
Methacrifos	12.89	207.9 -> 180.1	5	207.9 -> 93.0	10
Naled	16.83	108.9 -> 79.0	5	144.9 -> 109.0	15
Parathion-methyl	22.82	262.9 -> 109.0	10	232.9 -> 109.0	10
Procymidone	26.36	96.0 -> 67.1	10	96.0 -> 53.1	15
Pyridaben	32.59	147.2 -> 117.1	20	147.2 -> 132.2	10
Tecnazene	14.57	260.9 -> 203.0	10	214.9 -> 179.0	10
Terbufos	18.55	230.9 -> 175.0	10	230.9 -> 129.0	20
Terbufos sulfone	26.29	152.9 -> 96.9	10	198.9 -> 96.9	20
Tetramethrin-1	30.51	164.0 -> 107.1	10	164.0 -> 77.1	25
Tetramethrin-2	30.70	164.0 -> 107.1	10	164.0 -> 77.1	25
Vinclozolin	22.18	187.0 -> 124.0	20	197.9 -> 145.0	15

Table S5. Retention times and MRM parameters of phthalates for GC-MS/MS.

Phthalate	t_R(min)	MRM1	CE1 (ev)	MRM2	CE2 (ev)
Benzyl butyl phthalate	14.56	149 -> 65	20	149 -> 93	15
Bis(2-butoxyethyl) phthalate	15.88	149 -> 65	20	148.9 ->121.1	10
Bis(2-ethoxyethyl) Phthalate	12.17	104 -> 76	10	149->65	20
Bis(2-ethylhexyl) phthalate	16.71	167-> 149	10	148.9-> 120.9	10
Bis(2-methoxyethyl) phthalate	11.23	104->76.1	10	104 -> 49.8	25

Bis(4-methyl-2-pentyl) phthalate	11.90	167->149	10	148.9-> 120.9	10
Dibenzyl phthalate	19.42	107->79.1	10	91.1-> 65.1	15
Dibutyl phthalate	10.93	149-> 65	20	149-> 93	15
Dicyclohexyl phthalate	16.51	167->121	20	249->149	10
Diethyl phthalate	8.61	177->149	10	149-> 65	20
Dihexyl phthalate	14.41	149-> 65	20	148.9->120.9	10
Diisobutyl phthalate	10.27	149-> 65	20	149 -> 93	15
Diisodecyl phthalate	23.73	149->65	30	307->149.1	10
Diisononyl phthalate	22.09	149->65	25	293-> 149	10
Dimethyl phthalate	7.74	163-> 77	20	163-> 133	10
Dinoctyl phthalate	18.97	149 -> 65	20	149 -> 93	15
Dinonyl phthalate	23.53	149->93	15	148.9->120.9	20
Dipentyl phthalate	12.51	149-> 65	20	148.9-> 120.9	10

Table S6. The detection rates of Pb and Cd in conventional and hydroponic produced vegetable samples.

Vegetable	Heavy metal	Detection rate in conventional samples (%)	Detection rate in hydroponic samples (%)
lettuce	Pb	80.8	46.9
	Cd	84.6	31.2
celery	Pb	100	100
	Cd	100	73.3
tomato	Pb	72.7	70.0
	Cd	77.3	50.0
cucumber	Pb	65.2	78.9
	Cd	65.2	57.9
